# An ultra-high density linkage map and QTL mapping for sex and growth-related traits of common carp (*Cyprinus carpio*)

**DOI:** 10.1038/srep26693

**Published:** 2016-05-26

**Authors:** Wenzhu Peng, Jian Xu, Yan Zhang, Jianxin Feng, Chuanju Dong, Likun Jiang, Jingyan Feng, Baohua Chen, Yiwen Gong, Lin Chen, Peng Xu

**Affiliations:** 1 College of Life Sciences, Shanghai Ocean University, Shanghai, 201306, China; 2 Beijing Key Laboratory of Fishery Biotechnology, Centre for Applied Aquatic Genomics, Chinese Academy of Fishery Sciences, Beijing, 100141, China; 3 Henan Academy of Fishery Science, Zhengzhou, 450044, China; 4 College of Fishery, Henan Normal University, Xinxiang, 453007, China; 5 College of Ocean and Earth Sciences, Xiamen University, Xiamen 361102, PR China

## Abstract

High density genetic linkage maps are essential for QTL fine mapping, comparative genomics and high quality genome sequence assembly. In this study, we constructed a high-density and high-resolution genetic linkage map with 28,194 SNP markers on 14,146 distinct loci for common carp based on high-throughput genotyping with the carp 250 K single nucleotide polymorphism (SNP) array in a mapping family. The genetic length of the consensus map was 10,595.94 cM with an average locus interval of 0.75 cM and an average marker interval of 0.38 cM. Comparative genomic analysis revealed high level of conserved syntenies between common carp and the closely related model species zebrafish and medaka. The genome scaffolds were anchored to the high-density linkage map, spanning 1,357 Mb of common carp reference genome. QTL mapping and association analysis identified 22 QTLs for growth-related traits and 7 QTLs for sex dimorphism. Candidate genes underlying growth-related traits were identified, including important regulators such as *KISS2, IGF1, SMTLB, NPFFR1* and *CPE*. Candidate genes associated with sex dimorphism were also identified including *3KSR* and *DMRT2b*. The high-density and high-resolution genetic linkage map provides an important tool for QTL fine mapping and positional cloning of economically important traits, and improving common carp genome assembly.

Common carp (*Cyprinus carpio*) is one of the most important fishes that widely cultured in the world, which is not only an important food fish with an annual production of over 3 million metric tons^1^, but also an important ornamental fish due to the various skin colors and scale patterns in some strains such as koi, Oujiang color carp, Hebao red carp, *etc*. Common carp is a traditional aquaculture species which has been cultured in both Europe and East Asia for more than 2000 years. Due to its economical importance, common carp has been intensively studied on its physiology, development, immunology, disease control, genetic breeding, and transgenic manipulation. Besides, it is also widely used as model fish in various studies on ecology, environmental toxicology and evolution. In the past two decades, various genome resources and genetic tools have been developed to facilitate genetic improvement and breeding programs, including thousands of microsatellite and single nucleotide polymorphism (SNP) markers^2^,^3^, multiple versions of linkage maps^4^,^5^,^6^,^7^, BAC-based physical map^8^,^9^,^10^, expression sequence tags (ESTs) and transcriptome sequences^11^,^12^, cDNA microarrays^13^, SNP genotyping array^14^, *etc*. Recently, the genome of Songpu mirror carp, a strain derived from European subspecies (*C. carpio carpio*) of common carp, have been completely sequenced, providing the first reference genome for common carp genetic and genomic studies^15^.

Linkage maps are essential tools for genomic and genetic studies, as well as for genetic breeding of economically important species. In the past decades, a number of linkage genetic map had been constructed using various molecular genetic markers for many aquaculture species^16^,^17^, including many important teleost species such as catfish^18^,^19^, tilapia^20^,^21^, carps^5^,^6^,^22^, Asian seabass^23^,^24^, Japanese flounder^25^,^26^, Atlantic salmon^27^,^28^, rainbow trout^29^,^30^ and many others. High quality genetic linkage maps provide framework for quantitative trait loci (QTLs) localization, and facilitate genetic assistant selection and breeding in many aquaculture species. For instance, growth-related traits have been mapped and well-studied in many teleost fishes, such as rainbow trout^31^, Asian seabass^32^, salmons^33^; lymphocystis disease resistant traits in Japanese flounder have been successfully mapped and applied to marker-assistant breeding^34^,^35^; sex-determination traits have been localized in the genetic map by QTL mapping approaches in tilapia^36^, halibut^37^ and smooth half tongue sole^38^ and so on. Common carp genetic research community has constructed a number of linkage maps in the past decades. The first common carp linkage map was constructed based on 262 Radom Amplified Polymorphic DNA (RAPD) and Simple Sequence Repeat (SSR) markers in 2000^4^. Thereafter, many linkage maps have been constructed with more and more SSR and SNP markers based on different mapping families. These linkage maps have been widely used for QTLs mapping of many important traits on chromosomes, providing potentials for positional cloning of these traits. In the past decades, the QTLs of growth rate, body shape, swimming ability, meat quality and so on have been successfully mapped in common carp^6^,^7^,^39^,^40^,^41^,^42^. In addition, the linkage maps constructed using sequence-based markers can serve as a frame for comparative genomic analysis for understanding chromosomal organization and genome evolution. As an instance, the dense linkage map with 1,209 BAC-derived SSR and SNP markers had been used for comparative genomic study with zebrafish genome, which demonstrated that 50 common carp chromosomes are homologous with 25 zebrafish chromosomes. The evidences confirmed the “2 to 1” relationship on chromosomes of two species and revealed the tetraploid manner of common carp genome^6^. The high density sequence-based linkage map can provide the chromosome frame and “anchors” for genome sequence assembly and scaffolding, and ultimately construct the complete genome map. The latest linkage map comprising around 4,200 markers has been used for genome and genetic map integration and mapped 54% of common carp genome sequences onto the chromosomes^15^, generating the first reference genome on 50 chromosomes.

Due to the importance of linkage map in genomic and genetic studies, a linkage map with higher density and resolution is eagerly desired. However, it is always big challenge to obtain such a large number of genetic markers and genotype in relative large mapping families. The high throughput SNP genotyping array, Carp 250 K SNP array, has been recently developed based on Affymetrix Axiom platform^14^, providing an affordable tool for ultra-high density linkage map construction. The 250,000 high quality SNPs on the array are evenly selected from common carp reference genome with an average interval of 6.6 kb, providing the dense “molecular ruler” for genome scale genotyping. Therefore, we decided to use the powerful genotyping tool and construct high-density linkage genetic map for common carp.

Herein, we report the construction of an ultra-high density and high resolution linkage genetic map with over 28,194 SNP markers, which is the highest density genetic linkage map for common carp so far. Comparative genomic analysis with zebrafish and medaka genomes provides us new insights of common carp genome evolution. We also identified QTL loci for growth-related traits and sex dimorphism on the linkage map. The candidate genes were then recognized from the genome regions of QTL intervals. The high density linkage map provides a powerful tool for QTL fine mapping and association study of economically important traits, as well as a framework for improving reference genome of common carp.

## Results

### SNP marker filtration

SNP genotypes were obtained from 108 samples from the YRC mapping family including 2 parents and 119 progeny. A total of 199,233 SNP markers were used after assessment of genotyping quality and polymorphism in the mapping family. There were 13 progeny samples removed due to the low genotype calling rate (<95%). After further filtration with more stringent condition (SNPs calling rate greater than 95% and minor allele frequency (MAF) greater than 5%), a total of 87,323 SNP markers were retained for further analysis (Supplementary Fig. S1). We further selected 72,456 SNP markers based segregation distortion and non-Mendelian inheritance (P < 0.001) for further linkage analysis and mapping.

### Linkage mapping

As shown in supplementary Fig. S1, sex specific linkage maps were first constructed based on SNP markers that were heterozygous only in female parent (AB × AA or AB × BB, 26,918 SNPs) or male parent (AA × AB or BB × AB, 27,157 SNPs). To provide anchor markers for female-specific map and male-specific map integration, 510 SNP markers that were heterozygous in both parents (AB × AB with 1:2:1 segregation) were also included for linkage mapping. Thereafter, we used 27,428 SNP markers and 27,667 SNP markers for female-specific map and male-specific map construction, respectively. The female-specific genetic map consisted of 14,785 SNPs on 7,404 distinct positions in 50 LGs with a total genetic length of 7586.51 cM (Supplementary Fig. S2). The genetic length of each LG ranged from 54.27 cM (LG38) to 339.13 cM (LG11) with an average length of 151.73 cM (Supplementary Table S1). The inter-locus distance estimated based on the unique marker positions ranged from 0.77 cM in LG39 to 1.55 cM in LG38 with an average inter-locus distance of 1.02 cM in female-specific map. The male-specific genetic map consisted of 13,910 SNPs on 7,347 distinct positions in 50 LGs with a total genetic length of 8094.31 cM (Supplementary Fig. S3). The genetic length of each LG ranged from 75.77 cM (LG11) to 263.63 cM (LG32) with an average length of 161.89 cM (Supplementary Table S1). The inter-locus distance estimated based on the unique marker positions ranged from 0.85 cM in LG31 to 1.84 cM in LG27 with an average inter-locus distance of 1.10 cM in male-specific map.

**Figure 1:**
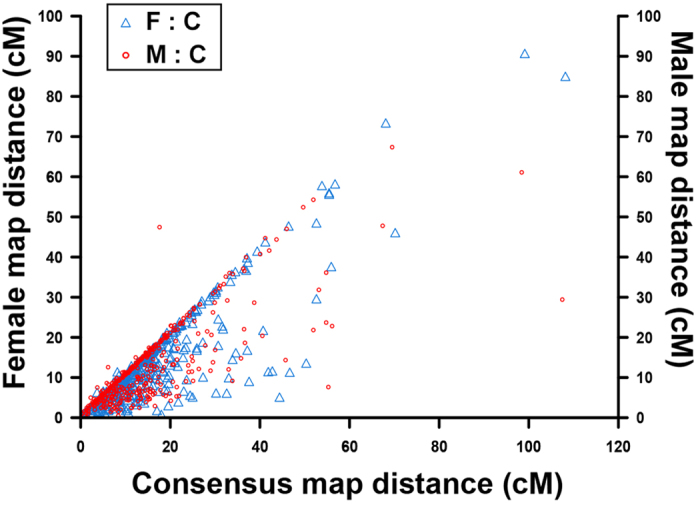
Recombination rate of shared markers between sex-specific maps and consensus map of Yellow River carp. This diagram was constructed using the recombination rate of 501 shared SNPs in sex-specific maps and consensus map. The X-axis stands for shared marker interval on the consensus map, and the left Y-axis represents shared marker interval on female map and right Y-axis represents the shared marker interval on male map. The triangles and circles represent shared marker interval ratio between female map and consensus map (F:C ratio), and between male map and consensus map (M:C ratio), respectively.

Overall, the female-specific map retained 57 more distinct loci, but was 507 cM shorter than male-specific map. The female-to-male LG length ratio ranged from 0.21 (LG38) to 4.48 (LG11) with an average ratio of 0.94. The average female-to-male ratio of inter-locus distance is 0.93, which suggests similar marker composition and linearity of both sex-specific maps. The genetic length of intervals on LGs reflects the recombination rates. A total of 501 shared SNP markers (heterozygous in both parents with 1:2:1 segregation) had been mapped in both female- and male-specific linkage maps, respectively. To better assess similarity and consistency of maps in both sexes, we calculated marker intervals of those shared SNP markers on each LGs of both sex-specific maps. The female to male ratios for the recombination rates of shared markers for each LGs ranged from 0.58 (LG32 and LG38) to 2.08 (LG15) with an average of 1.04 (Fig. 1, Supplementary Table S1). The recombination rate ratios of female-specific map were slightly higher than that of male-specific map. However, no significant difference was identified from both maps (t-test, p > 0.05). The evidence further revealed high similarity and collinearity of both sex-specific maps for common carp, which are critically important for generating a consensus map with high accuracy and quality.

**Figure 2:**
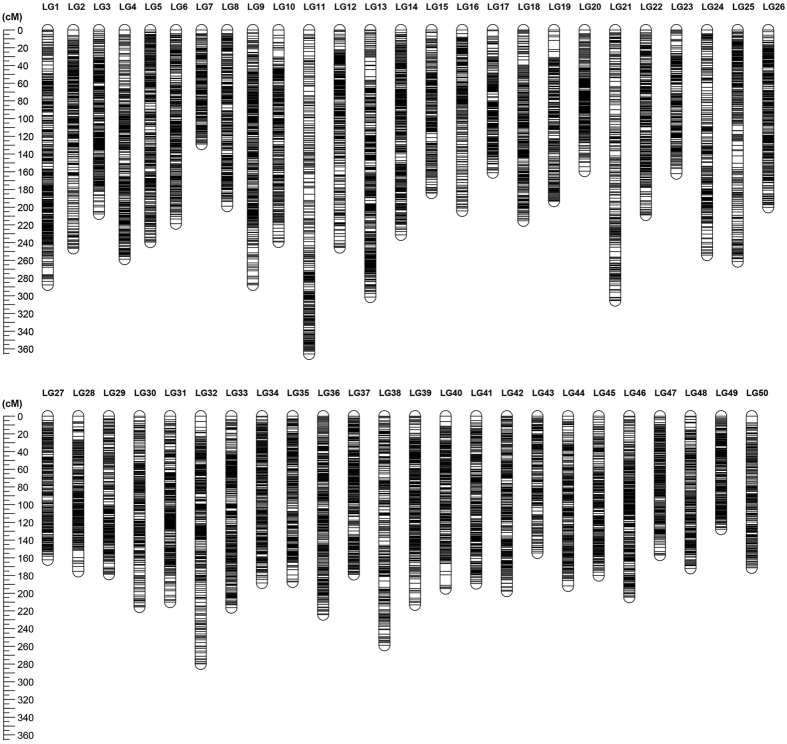


**Figure 3:**
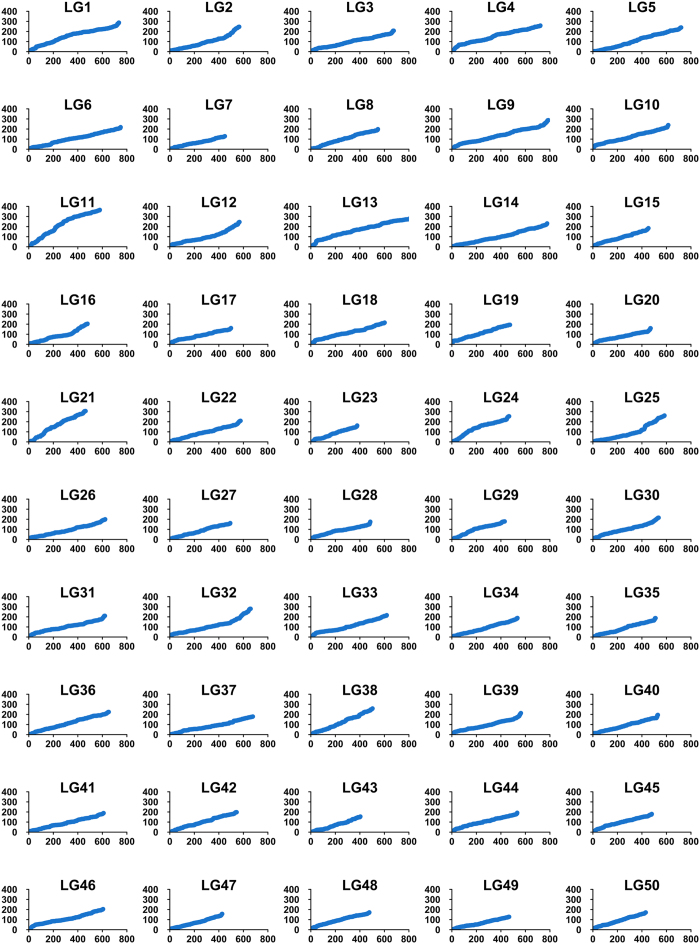
The patterns of marker distribution on each linkage group. The X-axis represents marker orders on each linkage group. The Y-axis represents SNP marker position (cM) on each linkage group.

Both sex-specific genetic maps were integrated into a consensus map based on the 501 shared SNP markers (Fig. 2). As summarized in Table 1, the consensus genetic map was comprised of 28,194 SNPs, including 14,284 female-specific markers, 13,409 male-specific markers and 501 shared markers, on 14,146 distinct positions in 50 LGs with a total genetic length of 10595.94 cM. The genetic length of each LG ranged from 127.69 cM (LG49) to 365.66 cM (LG11) with an average length of 211.92 cM (Table 1). The average locus intervals ranged from 0.58 cM in LG49 to 1.36 cM in LG21 with an overall average locus interval of 0.75 cM. The overall average marker interval was 0.38 cM. As shown in Fig. 3, SNP markers distribution on each LGs were examined, which illustrated even distribution of markers on the consensus map with some exceptions at terminal and middle regions of LGs.

### Genome scaffold anchoring and comparative genomics

The high-density, high-resolution genetic linkage map was comprised of 28,194 SNPs, which were approximate 7 fold more markers comparing with previous linkage map that used for Songpu mirror carp genome assembly^15^. Thus, the linkage map provided a new chromosome framework with more anchor points for whole genome sequences assembly and map integration. A total of 11,025 SNPs had been successfully mapped on genome sequences and anchored a total of 2,818 scaffolds representing 1,357 Mb onto the high-density linkage map, which was much longer than previously integrated genome regions (~892 Mb). Each chromosome comprised of an average genome region of 27.15 Mb, with a range from 12.95 Mb to 47.05 Mb (Supplementary Table S2 and S3).

**Figure 4:**
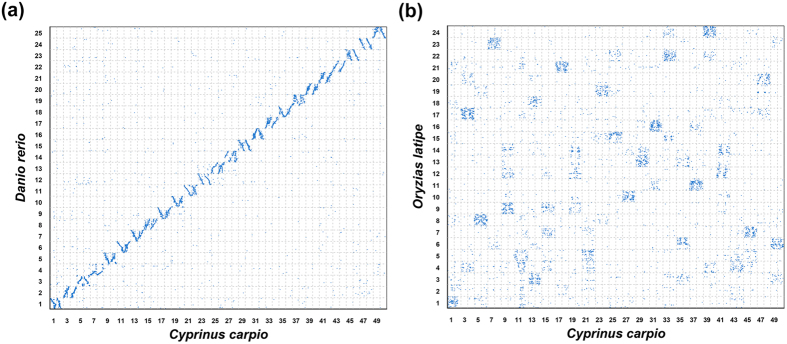
Genomic comparisons (a) between common carp and zebrafish, and (b) between common carp and medaka.

The high-density linkage map also provided new chromosome framework for comparative genomic studies with closely related model fishes. We extracted the genome sequences surrounding the SNPs and compared with protein sequences of zebrafish and medaka. A total of 10,021 1:1 best orthologues between common carp and zebrafish were identified. Of these orthologues, 9,947 were distributed on zebrafish chromosomes and only 74 were left on scaffolds. Hence, there were 200.4 orthologues on each common carp LG and 397.9 orthologues on each zebrafish chromosome on average, respectively. The Oxford plot was drawn based on the orthologous pairs between two species. As shown in Fig. 4, a perfect 2:1 chromosome relationship was observed between common carp and zebrafish genomes owing to the tetraploidized genome of common carp. Of the 9,947 orthologous pairs, vast majority pairs (9,241 pairs, 93%) had been mapped on those paired chromosomes of common carp and zebrafish, and built perfect chromosome-scale syntenies. The results demonstrated that the new linkage map of common carp was not only high density and high resolution map, but also high accurate map. Similarly, comparative genomic analysis was also performed between common carp and medaka genomes. A total of 7,467 1:1 best orthologues pairs were identified. Of these orthologues pairs, 6,887 were distributed on medaka chromosomes and 580 were left on scaffolds. As shown in Fig. 4, many syntenic blocks were built between two genomes, suggesting high similarity between two chromosomes. We also identified significant 2:1 chromosome relationship between common carp and medaka, for example, LG 5/6 vs. Chr 8, LG7/8 vs. Chr23, LG17/18 vs. Chr. 21, *etc*. Unlike common carp and zebrafish syntenies, we could not identified significant collinearity in the whole chromosome scale, which suggested that intensive chromosome rearrangements had occurred post the divergence of medaka and cyprinids.

### QTL mapping and association analysis of growth traits

Pairwise comparisons among three growth traits (BW, BL and CW) using Pearson’s correlation revealed that correlation coefficients of BW/BL, BW/CW and BL/CW were 0.845, 0.930 and 0.679, respectively (Supplementary Table S4). The correlation coefficients suggested that BW was highly correlated with BL, and CW was very highly correlated with BW, while CW was only moderately correlated with BL. Although QTL mapping of three growth-related traits showed that they exhibited similar QTL landscape, we did identified significant differences among QTL profiles of three traits, implying different genetic basis underlying these growth-related traits. As an alternative approach, we performed association analysis between SNP genotypes and growth phenotypes, which also revealed similar distribution patterns to that of QTL mappings. Integrating results from both approaches, we have more confidence to identify SNP loci that significantly associate with three growth-related traits.

**Figure 5:**
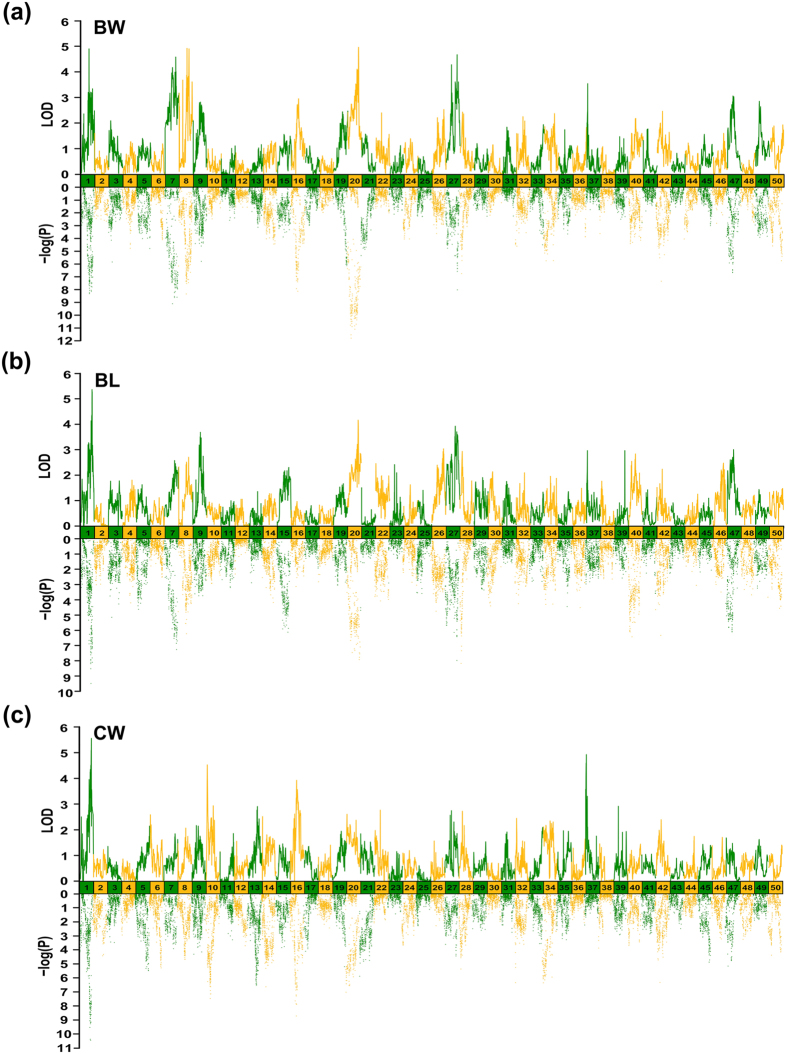
QTL mapping and associate analysis of growth-related traits in Yellow River carp. Significant regions were identified for (**a**) body weight (BW), (**b**) body length (BL) and (**c**) carcass weight (CW).

A total of 22 QTL regions associated with growth were identified based on chromosome-wide LOD significance with P < 0.01. We identified 14 QTL regions including 115 SNP loci for BW, distributing on five LGs including LG1, LG7, LG8, LG20 and LG27 (Fig. 5a and Table 2). The most significant QTL qBW20c located on LG20 at 118.65–122.51 cM presented the highest LOD value of 4.97, explaining 15.5% of the total phenotypic variations. The QTL qBW1 located on LG1 at 169.34–169.78 cM explained the highest percentage of the total phenotypic variations (45.1%) with a LOD value of 4.91. For BL, we identified four QTL regions including 19 SNP loci, distributing on three LGs including LG1, LG20 and LG27 (Fig. 5b and Table 2). The most significant QTL qBL1 located on LG1 at 238.65–239.51 cM presented the highest LOD value of 5.37, explaining 17.2% of the total phenotypic variations. The QTL qBL27b located on LG27 at 123.07–123.47 cM explained the highest percentage of the total phenotypic variations (17.3%) with a LOD value of 3.71. Four QTL regions were identified for CW that distributed on four LGs (LG1, LG10, LG16 and LG37) harboring 46 significant SNP loci (Fig. 5c and Table 2). The most significant QTL qCW1 located on LG1 at 236.09–242.2 cM presented the highest LOD value of 5.56, explaining 21.9% of the total phenotypic variations. The QTL qCW37 located on LG37 at 4.94–8.97 cM explained the highest percentage of the total phenotypic variations (38.8%) with a LOD value of 4.93. Of the QTLs of three growth-related traits, we identified significant portions that either shared or overlapped across three traits. For example, BW and BL shared similar QTL regions on LG20 that contained qBW20c and qBL20, and region on LG27 that contained qBW27b and qBL27b. BL and CW shared similar QTL region on LG1 that contained qBL1 and qCW1. The overlapped QTL regions suggested that relatively high correlations did exist among three growth-related traits in common carp although they had significant differences on their QTL profiles.

### Candidate gene identification for growth-related traits

To further identify potential causative genes underlying growth traits, we screened reference genome and collected protein-coding genes from the QTL regions. A total of 294, 63 and 153 genes were identified for BW, BL and CW, respectively. Interestingly, we recognized 25 genes that shared in BW and BL, and 69 genes shared in BL and CW. However, we did not identify any shared genes in BW and CW, possibly due to high stringency of LOD threshold (p < 0.01) applied on both traits. The protein-coding genes of those significant QTL regions were annotated by comparing with protein databases.

We identified the only gene, receptor tyrosine protein kinase erbB-4 (*ERBB4*), from the most significant QTL region (qBW1) on LG1, which explained 45.1% of the total phenotypic variations. *ERBB4* has been reported as one of the important candidate genes associated with obesity and body mass trait in human and pig^43^,^44^. We did not identify any significance in this region for CW in linkage mapping and association analysis. Therefore, we speculate that ERBB4 may be directly associated with visceral fat deposition and subsequently effect BW. We also identified two candidate genes in genome regions of QTL qBW7b on LG7, encoding kisspeptin 2 (*KISS2*) and glycogen synthase (*GS*), respectively. Kisspeptin can activate gonadotropin-releasing hormone (GnRH) neurons in the hypothalamus and causes the release of GnRH, which constitutes the initial step in the hypothalamic–pituitary–gonadal (HPG) axis^45^. Kisspeptin signaling pathway has been reported that links with obesity and body mass^46^,^47^. *GS* is the key enzyme in glycogenesis that converts glucose to glycogen. Glycogen would be subsequently converted into fat when glycogen storage levels are excessed. Higher *GS* expression in human can accelerate glycogen storages and lead to obesity^48^. Hence, we suggested that both *KISS2* and *GS* were associated with body growth, energy deposition and fat accumulation in common carp. We also identified insulin-like growth factor 1 (*IGF1*) gene in the QTL qBW8a in LG8. *IGF1* is important constituent of growth hormone (GH)/IGF-I axis, which play important roles in the promotion of cell proliferation and the inhibition of cell death. IGF1 had been reported that associated with growth-related traits in various animals, e.g. mice^49^, chicken^50^, tilapia^51^. Interestingly, we identified another copy of *GS* gene in QTL qBW8a, which could be the paralogs of the one in QTL qBW7b on LG7. Both *GS* genes were likely derived from the WGD event retaining similar function on glycogen regulation. On LG20, we identified somatolactin beta (*SMTLB*) in QTL qBW20a. Somatolactin is a fish specific adenohypophyseal peptide hormone related to GH^52^,^53^. Thus, we speculate that *SMTLB* plays similar roles of GH in regulating growth and development. We further identified two neuropeptide FF receptor 1 (*NPFFR1*) genes in QTL qBW20b and qBW20c on LG20, respectively. NPFF1 is receptor of neuropeptide FF (NPFF) and RFamide-related peptide (RFRP), which are involved in control of feeding behavior both in invertebrates and in vertebrates^54^,^55^. Therefore, we suggested that *NPFFR1*s might be related to growth and body weight in common carp. We also observed genes that encode bone morphogenetic protein 10 (*BMP10*) and Bone morphogenetic protein receptor type-1B (*BMPR1B*) in QTL qBW20c. BMP signaling pathway is critical for bone and cartilage development, which also possibly associated with growth related traits in common carp.

For BL, we also identified *NPFFR1* and *BMP10* genes from QTL qBL20, which was overlapped with qBW20c on LG20. It suggested that *NPFFR1* and *BMP10* genes possibly contribute to both BW and BL of common carp. We also identified another candidate gene, carboxypeptidase E (*CPE*), from QTL qBL1 on LG1. Interestingly, the QTL qCW1 for CW covered QTL region qBL1 for BL. Therefore, *CPE* were significantly associated with both traits. Carboxypeptidase E is a peptide processing enzyme, involved in cleaving numerous peptide precursors, including neuropeptides and hormones involved in appetite control and glucose metabolism^56^,^57^. *CPE* mutations induced obesity in human and mice 24,25. We proposed that *CPE* contributes to body growth in common carp.

For CW, we further recognized two fibroblast growth factor-binding protein (FGFBP) genes, *FGFBP1* and *FGFBP2*, from QTL qCW1 that were tandemly located on LG1 and fibroblast growth factor receptor 1 (*FGFR1*) gene from QTL qCW16 on LG16 which play important roles in embryogenesis, cellular differentiation, and proliferation. *FGFBP1* and *FGFBP2* genes had been reported that associated with carcass and meat quality traits in chickens^58^,^59^, and FGFR1 gene had been reported that correlated with fetal weight in ovine^60^. So these genes were considered that associated with CW in common carp as well. Strikingly, we identified two copies of NPFFR1-like 2 (*NPFFR1L2*) genes that were tandemly located in QTL qCW10 on LG10, which was a distinctive QTL region from BW and BL. The two *NPFFR1L2* genes likely play similar roles to that of NPFFR1, and specifically associate with CW of common carp. The candidate genes for growth-related traits well supported by previous research were listed in Table 3.

### QTL mapping and association analysis of sex dimorphism

**Figure 6:**
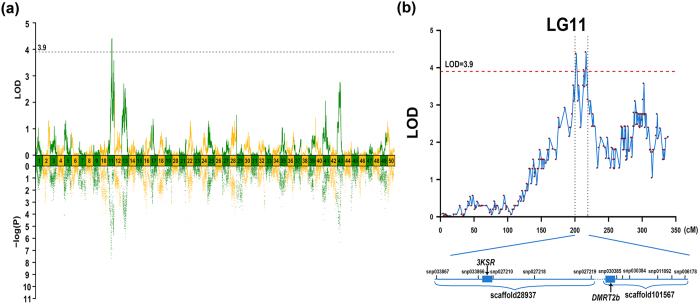
QTL mapping and associate analysis of sex dimorphism in Yellow River carp (a) among all linkage groups and (b) candidate regions with the QTL in LG11. The dashed line indicates the genome-wide significance threshold where LOD = 3.9. The vertical dashed lines indicate two QTL regions (qSD11a and qSD11b) that harbor candidate genes *3KSR* and *DMRT2B*.

We identified seven significant QTL regions (chromosome-wide p < 0.01) for sex determination on LG11, and LG43. As an alternative approach, we performed association analysis between SNP genotypes and sex phenotypes, which demonstrated almost identical distributions to that of QTL mapping (Fig. 6 and Table 2). Therefore, we confidently identify 55 SNP loci from the seven significant QTL regions. The most significant QTL qSD11b located on LG11 at 118.65–122.51 cM presented the highest LOD value of 4.42, explaining 19.7% of the total phenotypic variations. Its neighboring QTL qSD11a also explained 19.5% of the total phenotypic variations with a LOD value of 4.38.

We identified a protein-coding gene hydroxysteroid (17-Beta) dehydrogenase 7 (*HSD17B7*) that encodes 3-keto-steroid reductase (3KSR), which is also called 3-beta-hydroxysteroid 3-dehydrogenase (3β-HSD), in QTL qSD11a on LG11. 3β-HSD is one of the important enzymes in steroidogenesis pathway, which catalyzes the biosynthesis of progesterone from pregnenolone, 7α-hydroxyprogesterone from 17α-hydroxypregnenolone, androstenedione from dehydroepiandrosterone, and testosterone from androstenediol, *etc*. Therefore, 3β-HSD is essential for the biosynthesis of all classes of hormonal steroids, including progesterone, glucocorticoids, mineralocorticoids, androgens, and estrogens. Together with other regulating enzymes, 3β-HSD had been recognized as an important regulator for gonadal sex differentiation in tilapia^61^. Therefore, we speculated that *HSD17B7* was also significantly linked for gonadal sex differentiation in common carp. We further identified doublesex and mab-3 related transcription factor 2b (*DMRT2b*) gene in QTL qSD11b on LG11, which was the most significant QTL region for SD. *DMRT* genes are expressed in tightly restricted spatial patterns in association with the development of sex-specific organs, which associate with sex dimorphism in many animals^62^,^63^,^64^. Thus, we suggested that *DMRT2b* could potentially associate with sex determination in common carp as well. We also identified testis-specific serine/threonine-protein kinase 6 (*TSSK6*) gene from QTL qSD43c on LG43. TSSK6 had been reported that is essential for spermiogenesis and male fertility in mice^65^, and might be also associated with SD in common carp.

## Discussion

### Ultra-high density genetic map for common carp

Comparing with other genetic markers, SNP markers are the most abundant type of marker in the genome with high polymorphism. It is the most suitable markers for high density linkage map construction. However, it is used to be a big challenge to develop sufficient number of SNP markers and conduct cost-effective genotyping in a relative large mapping population, especially for many non-model species such as aquaculture species. With advances of the next-generation sequencing technologies, more and more reference genomes have been completely sequenced and assembled, and various sequence-based SNP genotyping technologies have been developed providing rapid and cost-effective high-throughput SNP genotyping platforms for linkage mapping. Recently, a number of high density linkage maps have been constructed for aquaculture species based on sequence-based SNP genotyping technologies, including Japanese flounder^26^, Asian sea bass^24^, sea cucumber^66^, white shrimp^67^, abalone^68^, *etc*, which are tremendous progress on genetic studies of these species. One major challenge with sequence-based SNP genotyping approaches is the relatively high rates of genotype errors and a large number of missing genotype data, especially for those non-model species without reference genome^69^,^70^. Genotyping errors and missing data would lead to incorrect map orders and inflation of map lengths, particularly as marker density increases^71^. For instance, the genetic sizes of initial map were over 1,000 cM per chromosome in zebrafish. After removal of genotyping errors, the genetic sizes were reduced to around 100 cM^72^. Therefore, genotyping accuracy and call rate are critically importance for high-density linkage map construction. Comparing with sequence-based SNP genotyping approaches, Affymetrix Axiom SNP genotyping platform has higher call rates (>99%) and higher accuracy without intensive bioinformatics analysis^14^. Recently, a high-density genetic linkage map was constructed based on Affymetrix Axiom SNP genotyping data for channel catfish, presenting the highest marker density with high accuracy among linkage maps of aquaculture species^19^. Despite the relative higher cost, we still chose common carp 250 K SNP array for SNP genotyping and constructed the high-density and high-accurate linkage genetic map for common carp, which provides a dense chromosome framework to correctly order the genome scaffolds and locate QTLs on chromosomes.

After genotyping quality control and polymorphism detection in the mapping family, we’ve got a total of 199,233 SNP markers to be used. In order to get a high-quality linkage map, we used higher stringency on the data filtration (missing value <5% and MAF >5%) than most of the high density linkage mapping studies^19^,^24^, and finally retained 72,456 SNP markers. The recombination rate comparison (Fig. 1) demonstrated that both sex-specific maps and the consensus map of Yellow River carp were high quality, high accurate genetic maps and were highly consistent with each others.

The commonly used mapping families for linkage genetic map construction include recombinant inbred lines (RIL), double haploid (DH), F2 and backcross (BC) families, which provide genetic segregation loci for linkage analysis. However, it is great challenge to construct RIL, DH families in teleost fish. It usually takes 5–6 years to construct F2 and BC mapping families and measure economical traits successfully. Therefore, we decided to construct genetic linkage map using F1 mapping family with double pseudo-testcross strategy, which was first proposed by Grattapaglia and Sederoff^73^ and has been successfully applied to genetic linkage map construction in many aquaculture species^74^. The Yellow River carp broodstock were derived from wild population in the Yellow river approximate 20 years ago, which still retains high level of heterozygosity. Hence, the F1 progeny displays substantial segregation and have many different types of segregation. We collected genotyping data of 199,233 SNP markers, which are sufficient to select backcross pattern in a 1:1 ratio as testcross loci, including 26,918 SNPs for female-specific map and 27,157 SNPs for male-specific map construction in this study. The large number of testcross loci would enable high density genetic map construction and QTL localization with high efficiency and accuracy.

The total length of the consensus linkage map is 10595.94 cM which is extremely longer than many other teleost species such as Asian seabass, grouper, Japanese flounder, channel catfish, large yellow croaker and so on. There could be several reasons related to the long genetic map. First of all, common carp is allotetraploid that had undergone a 4th round whole genome duplication (WGD). Therefore, common carp retained doubled chromosomes (n = 50) comparing with many other diploid teleost, and doubled the overall map length. Secondly, we used remarkably abundant SNP markers for the linkage mapping, which provided more chance to map markers into those marker absence regions, especially in the telomeric regions^75^. Subsequently, more recombination events could be recognized and enlarge the total length of the genetic map accordingly. Thirdly, the consensus map was constructed by merging two sex-specific maps using the approximation algorithm proposed by Wu *et al*.^76^, which may lead to map length increase in some cases^26^,^77^.

In this study, we took advantage of the common carp 250 K SNP genotyping array, collected high throughput genotyping data accurately and efficiently from a Yellow River carp mapping family, and constructed a high-density and high-resolution genetic map for common carp. To our best knowledge, this map is the highest linkage genetic map among all genetic map for common carp, which increased marker density by almost one order of magnitude comparing with previously constructed “high-density” genetic map. It is also ranked as one of the linkage genetic maps that retain highest genetic marker density in aquaculture species so far, providing valuable resource for fine mapping of important economic traits as well as for comparative genomics and genome assembly.

### Chromosome framework for genome scaffolding and map integration

The high-density linkage maps provide chromosome framework for genome assembly validation. For example, an high density genetic linkage map had been constructed for genome assembly validation in oyster, which revealed widespread errors on whole genome assembly^78^. We used the high-density genetic linkage map as a new chromosome framework and anchored previous published common carp (Songpu mirror carp, subspecies *C. carpio carpio*) genome sequences onto the chromosome. A total of 1,357 Mb of the genome had been successfully integrated with the high-density linkage map, which significantly increased genome integrity. With the new chromosome framework, we also identified a significant portion of the scaffolds that were disagreed with marker position on the genetic linkage map (1,757 out of 2,818 mappable scaffolds were mapped onto two or more LGs), possibly as a result of assembly errors. An alternative reference genome employing new sequencing technologies and assembly algorithms would be an easy way to correct these errors. Therefore, we have initiated genome sequencing and *de novo* assembly of Hebao red carp (*C. carpio var.wuyuannensis*), which is representative variety of subspecies *C. carpio haematopterus.* The new reference genome has been assembled with scaffold N50 of 0.92 Mb. Therefore, we were able to anchor these scaffolds onto the high-density genetic linkage map and compared with previously published reference genome. A total of 26,308 SNPs had been successfully mapped on new reference genome and anchored a total of 1,236 Mb onto the high-density linkage map. Each chromosome comprised of an average genome region of 24.73 Mb, with a range from 16.98 Mb to 35.62 Mb (supplementary Table S5 and S6). The number of scaffolds that were anchored to different LGs had been significantly reduced comparing with previous reference genome (124 out of 2,963 mappable scaffolds were mapped onto two or more LGs), demonstrating the higher accuracy on connectivity and contiguity of the new reference genome. The new reference genome is expected to be completed soon, providing more genome resources for further understanding the essence of common carp.

### Growth-related QTLs and candidate genes

The great efforts have been devoted on growth-related traits in aquaculture species, and common carp is one of the most investigated species. QTL mapping and association studies on growth-related traits have been intensively performed on common carp in multiple research groups^7^,^39^,^40^,^41^,^42^,^79^. However, it was great difficulty for QTL fine mappings and causative gene identification due to the absence of a reference genome and high throughput genotyping data. In this study, we were able to take advantages of high-throughput SNP genotyping array and two reference genomes, providing new insights into growth-related traits and their related genes. We identified a group of candidate genes underlying growth-related phenotypes. As we described in the results, most of these growth-relate genes are important components on HPG axis and GH/IGF-I axis that regulate development, cell-proliferation, energy metabolism and growth. The results also provided a bunch of growth traits associated SNP markers, which will facilitate marker-assistance parental fish selection for our ongoing selective breeding program of Yellow River carp. In conclusion, we performed high-throughput SNP genotyping on a mapping family of Yellow River carp, and constructed a high-density, high-resolution genetic linkage map for common carp. The new genetic map processes the highest marker density among the genetic maps of common carp. We have accurately mapped growth-related QTLs and sex dimorphism traits onto the genetic map, and identified functional genes underlying these traits. With the new chromosome framework, we anchored genome scaffolds onto the genetic map with much higher integration rates. Comparative genomic analyses have been conducted among common carp and closely related model species, providing new insights into genome evolution of common carp. The high-density and high-resolution genetic linkage map provides an important tool for QTL fine mapping of economically important traits as we demonstrated, as well as for genome assembly improvement. The results of QTL fine mapping of growth and sex dimorphism will be also used in the future genetic breeding programs of common carp.

## Material and Methods

### Ethics Statement

This study was approved by the Animal Care and Use committee of Centre for Applied Aquatic Genomics at Chinese Academy of Fishery Sciences. The methods were carried out in accordance with approved guidelines.

### Mapping family and DNA extraction

Yellow River carp (YRC) is a typical strain of Eastern Asian subspecies (*C. carpio haematopterus*) of common carp. A F1 full-sib family of YRC was constructed at Breeding Station of Henan Academy of Fishery Research, Zhengzhou, Henan province, China. Approximate 3,000 offspring were raised in a 2000-m^2^ pond and fed four times daily under standard feeding regime (3% feed/body weight ratio daily). The oxygen level was maintained at 3 mg/L or above. A total of 119 progeny were randomly collected 18 months post hatch from the mapping family for genotyping and trait measure. Growth-related traits including body weight (BW), body length (BL) and carcass weight (CW) were measured. CW refers to the weight of an individual after removing all the internal organs, gills and scales. Sex of each individual was determined after dissection. Genomic DNA was extracted from blood using DNeasy 96 Blood & Tissue Kit (Qiagen, Shanghai, China). After quantification by Nanodrop-1000 spectrophotometer (Thermo Scientific, Wilmington, DE, USA) and integrity examination by agarose gel electrophoresis, DNA samples were stored at −20 °C for further experiments.

### SNP genotyping

DNA samples used for genotyping were diluted to a final concentration of 50 ng/μl and genotyped at GeneSeek (Lincoln, NE, USA) using the common carp 250 K SNP array^14^. Affymetrix CEL files were analysed using Affymetrix Genotyping Console software (version 4.0) for quality control analysis and SNP genotype calling using the Affymetrix Axiom GT1 algorithm. Dish value was set as default and SNPs with call rates greater than 95% were collected for further analysis. The CHP files generated from Affymetrix Genotyping Console were then extracted and converted to Ped/Map format that served as input files to PLINK software for further analysis. All individuals with missing genotypes >5% and SNPs with missing genotypes >5%, minor allele frequency <5% were removed using the “--mind”, “--geno” and “--maf” parameters. In-house perl scripts were used for data cleaning. Only the SNPs that are heterozygous in at least one parent and conform to Mendelian inheritance were used for further linkage analysis.

### Linkage map construction

The double pseudo-test cross strategy was employed for linkage analysis. SNPs were divided into three categories according to their segregation patterns: ABxAA or ABxBB (1:1 segregation only in female parent), AAxAB or BBxAB (1:1 segregation only in male parent), and ABxAB (1:2:1 segregation in both parents). Both female- and male-specific linkage maps were constructed by using JoinMap 4.1^80^ with “CP” type population, which is designed to handle F1 population data containing various genotype configurations. A LOD threshold of 7.0 was used for assigning markers into different linkage groups (LGs). Recombination frequencies of markers on the same LG were converted into map distances (cM) through the maximum likelihood (ML) algorithm. The consensus map was then established using the MergeMap^76^ (http://www.mergemap.org/) by integrating sex-specific maps through shared markers. All genetic linkage maps were drawn using MapChart 2.2^81^. A schematic pipeline is shown in Supplementary Fig. S1.

### Genome integration and comparative genomics

The SNP sequences (71-bp in length) on the consensus map of YRC were mapped to reference genome of Songpu mirror carp^15^ and unpublished reference genome of Hebao red carp by using BLASTN. The SNPs with an alignment length ≧ 70 bp and gap length <1 bp were considered as accurate mapping. The scaffolds were therefore mapped to LGs according to their best match. The position and orientation of scaffolds with at least two SNP anchors were determined. However, those scaffolds contain only one SNP anchor were unable to determine their orientation on LGs.

The high density genetic map of YRC allowed us to perform accurate comparative genomic study with closely related model species such as zebrafish and medaka. Of the 250,000 high quality SNPs, over 147,000 SNPs were collected from transcriptome sequences. Therefore, majority of SNP markers on the consensus map represent gene sequences and are very useful for comparative genomic analysis. The zebrafish and medaka protein sequences were downloaded from the Ensembl databases^35^,^72^. The genome sequences surrounding the SNP loci (1,001 bp with SNP loci in the center) were extracted from reference genome by using in-house perl scripts. BLASTx searches were performed using SNP sequences against protein sequences of zebrafish and medaka with e-value cutoff of e^−10^.

### QTL mapping of growth-related traits and sex dimorphism

Pearson’s correlations among three growth-related traits (BW, BL and CW) were performed in all progeny. The Student’s t-test was used to establish if the correlation coefficient is significantly different from zero (p-value ≤ 0.05). QTL mapping analysis was performed for growth-related traits and sex dimorphism (SD) trait using MapQTL 6 software package^80^ with “composite interval mapping” and “restricted multiple QTL model (MQM) mapping” algorithms. LOD score significance thresholds were determined by 1,500-permutation tests for each trait. QTL with LOD scores exceeding the chromosome-wide LOD threshold at P < 0.01 or the genome-wide LOD threshold at P < 0.05 were considered as significant. Association analyses between genotypes and traits were performed using PLINK with a simple linear regression model of phenotype on genotype. Integrating the results from both QTL mapping and association analysis, we identified SNP loci that significantly correlated with the traits, and extracted candidate genes around these SNP loci from reference genome of common carp.

## Additional Information

**How to cite this article**: Peng, W. *et al*. An ultra-high density linkage map and QTL mapping for sex and growth-related traits of common carp (*Cyprinus carpio*). *Sci. Rep.* **6**, 26693; doi: 10.1038/srep26693 (2016).

## Supplementary Material

Supplementary Figures and Tables

Supplementary Dataset

## Figures and Tables

**Table 1:** Summary of the consensus linkage map of Yellow River carp.

LG	Consensus map						
	No. of SNPs	Female-specific makers	Male-specific markers	Shared markers	Distinct positions	Distance (cM)	Locus interval (cM)
1	735	397	328	10	390	287.65	0.74
2	566	154	402	10	296	246.46	0.84
3	676	374	292	10	346	207.55	0.60
4	722	302	410	10	344	258.71	0.75
5	720	371	339	10	367	239.39	0.65
6	749	277	462	10	308	218.61	0.71
7	449	235	204	10	218	128.84	0.59
8	547	406	131	10	271	198.89	0.74
9	787	383	394	10	406	287.69	0.71
10	615	368	237	10	319	239.30	0.75
11	579	438	131	10	298	365.66	1.23
12	568	192	366	10	301	245.62	0.82
13	839	456	373	10	425	301.52	0.71
14	777	357	410	10	371	231.32	0.63
15	453	238	205	10	237	184.24	0.78
16	479	246	222	11	231	204.16	0.89
17	499	258	231	10	235	161.08	0.69
18	602	276	315	11	299	215.38	0.72
19	474	265	199	10	234	193.31	0.83
20	471	243	218	10	230	159.38	0.70
21	463	387	66	10	226	305.47	1.36
22	577	266	301	10	289	208.94	0.73
23	378	254	114	10	196	162.15	0.83
24	467	314	147	6	230	254.10	1.11
25	584	361	213	10	277	261.36	0.95
26	623	301	312	10	330	200.20	0.61
27	493	366	117	10	228	162.26	0.71
28	485	253	221	11	250	175.37	0.70
29	431	239	183	9	223	178.60	0.80
30	536	202	324	10	252	215.60	0.86
31	621	214	397	10	321	209.99	0.66
32	659	205	443	11	354	279.86	0.79
33	619	246	363	10	327	216.27	0.66
34	535	343	182	10	310	188.50	0.61
35	511	257	244	10	265	187.26	0.71
36	652	311	331	10	333	224.20	0.68
37	677	341	327	9	310	178.88	0.58
38	503	66	427	10	249	258.84	1.04
39	563	266	287	10	309	213.24	0.69
40	530	271	248	11	247	194.81	0.79
41	610	357	243	10	271	189.06	0.70
42	545	225	310	10	277	197.61	0.72
43	404	227	167	10	196	154.55	0.79
44	533	299	224	10	256	191.82	0.75
45	480	187	283	10	261	180.10	0.69
46	606	290	305	11	313	204.40	0.66
47	427	241	175	11	239	156.87	0.66
48	477	305	162	10	243	171.91	0.71
49	467	221	236	10	221	127.69	0.58
50	431	233	188	10	217	171.27	0.79
Total	28194	14284	13409	501	14146	10595.94	0.75

**Table 2:** Genomic regions associated with growth-related traits and sex dimorphism in Yellow River carp.

Traits	QTL name	LG	CI (cM)	No. of SNPs	LOD	Permutation*	Exp%	Nearest marker
BW	qBW1	1	169.34–169.78	1	4.91	4.2	45.1	snp034657
	qBW7a	7	60.3–60.9	4	3.97	3.8	11.2	snp029272
	qBW7b	7	64.92–68.13	14	4.18	3.8	11.6	snp030451
	qBW7c	7	71.12–74.06	8	3.88	3.8	11.2	snp030717
	qBW7d	7	89.98–90.93	5	4	3.8	30.5	snp028769
	qBW7e	7	97.4–97.63	2	4.59	3.8	40.9	snp028874
	qBW7f	7	99.53–100.01	2	4.02	3.8	12.3	snp064916
	qBW8a	8	110.47–111.17	9	4.93	4.2	16.6	snp003905
	qBW8b	8	137.04–140.47	1	4.92	4.2	12.4	snp030579
	qBW20a	20	101.88–103.99	13	4.12	4	12.5	snp027051
	qBW20b	20	112.01–113.35	12	4.08	4	12.2	snp066338
	qBW20c	20	118.65–122.51	25	4.97	4	15.5	snp012238
	qBW27a	27	58.77–59.76	16	4.29	4	36	snp071640
	qBW27b	27	123.07–124.09	2	4.68	4	35.6	snp033523
BL	qBL1	1	238.65–239.51	8	5.37	5.3	17.2	snp024629
	qBL20	20	119.65–121.56	9	4.17	3.7	17	snp064795
	qBL27a	27	106.05–107.09	1	3.93	3.7	14.4	snp033534
	qBL27b	27	123.07–123.47	1	3.71	3.7	17.3	snp019515
CW	qCW1	1	236.09–242.2	21	5.56	4.7	21.9	snp015388
	qCW10	10	19.74–22.62	2	4.53	4.2	23.3	snp057007
	qCW16	16	90.08–90.22	10	3.93	3.9	14.2	snp005616
	qCW37	37	4.94–8.97	13	4.93	4.1	38.8	snp050105
SD	qSD11a	11	197.29–206.95	16	4.38	3.1	19.5	snp033867
	qSD11b	11	211.87–223.35	28	4.42	3.1	19.7	snp016532
	qSD11c	11	289.88–290.39	1	3.11	3.1	14.3	snp004413
	qSD11d	11	301.75–302.75	2	3.58	3.1	16.2	snp009064
	qSD43a	43	55.47–55.90	1	2.76	2.7	12.8	snp067977
	qSD43b	43	58.46–59.13	1	2.76	2.7	12.8	snp068014
	qSD43c	43	63.59–63.75	6	2.73	2.7	12.6	snp006435

Note: *Represents the chromosome-wide significance LOD threshold at P < 0.01.

**Table 3:** Summary of candidate genes for growth traits in Yellow River carp.

Traits	QTL name	LG	Scaffold ID	Gene name	Annotation
BW	qBW1	1	scaffold67929	*ERBB4*	receptor tyrosine protein kinase erbB-4
	qBW7b	7	scaffold101977	*KISS2*	kisspeptin 2,
				*GS*	glycogen synthase
	qBW8a	8	scaffold80123	*IGF1*	insulin-like growth factor 1
				*GS*	glycogen synthase
	qBW20a	20	scaffold98823	*SMTLB*	somatolactin beta
	qBW20b	20	scaffold33667	*NPFFR1*	neuropeptide FF receptor 1
	qBW20c	20	scaffold38649	*NPFFR1*	neuropeptide FF receptor 1
				*BMP10*	bone morphogenetic protein 10
BL	qBL1	1	scaffold33901	*CPE*	carboxypeptidase E
	qBL20	20	scaffold38649	*NPFFR1*	neuropeptide FF receptor 1
				*BMP10*	bone morphogenetic protein 10
CW	qCW1	1	scaffold33901	*CPE*	carboxypeptidase E
				*FGFBP1*	fibroblast growth factor-binding protein 1
				*FGFBP2*	fibroblast growth factor-binding protein 2
	qCW10	10	scaffold87389	*NPFFR1L2*	NPFFR1-like 2
				*NPFFR1L2*	NPFFR1-like 2
