# Erratum: An ultra-high density linkage map and QTL mapping for sex and growth-related traits of common carp (*Cyprinus carpio*)

**DOI:** 10.1038/srep30101

**Published:** 2016-07-22

**Authors:** Wenzhu Peng, Jian Xu, Yan Zhang, Jianxin Feng, Chuanju Dong, Likun Jiang, Jingyan Feng, Baohua Chen, Yiwen Gong, Lin Chen, Peng Xu

Scientific Reports 6: Article number: 2669310.1038/srep26693; published online: 05262016; updated: 07222016

In the original version of this Article, Affiliation 3 was omitted for Wenzhu Peng. The correct affiliations are listed below:

College of Life Sciences, Shanghai Ocean University, Shanghai, 201306, China.

Beijing Key Laboratory of Fishery Biotechnology, Centre for Applied Aquatic Genomics, Chinese Academy of Fishery Sciences, Beijing, 100141, China.

College of Ocean and Earth Sciences, Xiamen University, Xiamen 361102, PR China

In addition, Affiliations 3, 4 and 5 were incorrectly listed as 5, 3 and 4 respectively.

These errors have now been corrected in the PDF and HTML versions of this Article.

