# Supplementary material for: An ultra-high density linkage map and QTL mapping for sex and growth-related traits of common carp (Cyprinus carpio)
Source: Sci Rep. 2016 May 26;6:26693. doi: 10.1038/srep26693 (PMC4880943; doi:10.1038/srep26693)
Supplement: Supplementary Figures and Tables [file srep26693-s1.doc]

**An ultra-high density linkage map and QTL mapping for sex and growth-related traits of common carp (*Cyprinus carpio*)**

Wenzhu Peng^1,2,†^, Jian Xu^2,†^, Yan Zhang^2,†^, Jianxin Feng^4^, Chuanju Dong^1,2^, Likun Jiang^1,2^, Jingyan Feng^1,2^, Baohua Chen^1,2^, Yiwen Gong^1,2^, Lin Chen^2,5^, Peng Xu^2,3,*^

^1^College of Life Sciences, Shanghai Ocean University, Shanghai, 201306, China

^2^Beijing Key Laboratory of Fishery Biotechnology, Centre for Applied Aquatic Genomics, Chinese Academy of Fishery Sciences, Beijing, 100141, China

^3^College of Ocean and Earth Sciences, Xiamen University, Xiamen 361102, PR China

^4^Henan Academy of Fishery Science, Zhengzhou, 450044, China

^5^College of Fishery, Henan Normal University, Xinxiang, 453007, China

^†^ These authors contributed equally to this work.

* Corresponding author: Peng Xu, E-mail: [xupeng@cafs.ac.cn](mailto:xupeng@cafs.ac.cn)

**Supplementary data:**

Supplementary Figure S1: The pipeline of linkage map construction.

Supplementary Figure S2: The female-specific linkage genetic map for Yellow River carp.

Supplementary Figure S3: The male-specific linkage genetic map for Yellow River carp.

Supplementary Table S1: Summary of the sex-specific linkage maps of Yellow River carp.

Supplementary Table S2: Integration of consensus linkage map and reference genome of Songpu mirror carp.

Supplementary Table S3: Details of physical map of Songpu mirror carp anchored to linkage map.

Supplementary Table S4: Correlation analysis of three growth-related traits in Yellow River carp.

Supplementary Table S5: Integration of the consensus linkage map and reference genome of Hebao red carp.

Supplementary Table S6: Details of physical map of Hebao red carp anchored to linkage map.

Supplementary dataset: All the dataset of the linkage maps in spreadsheets.

**Figure S1:** The pipeline of linkage map construction.


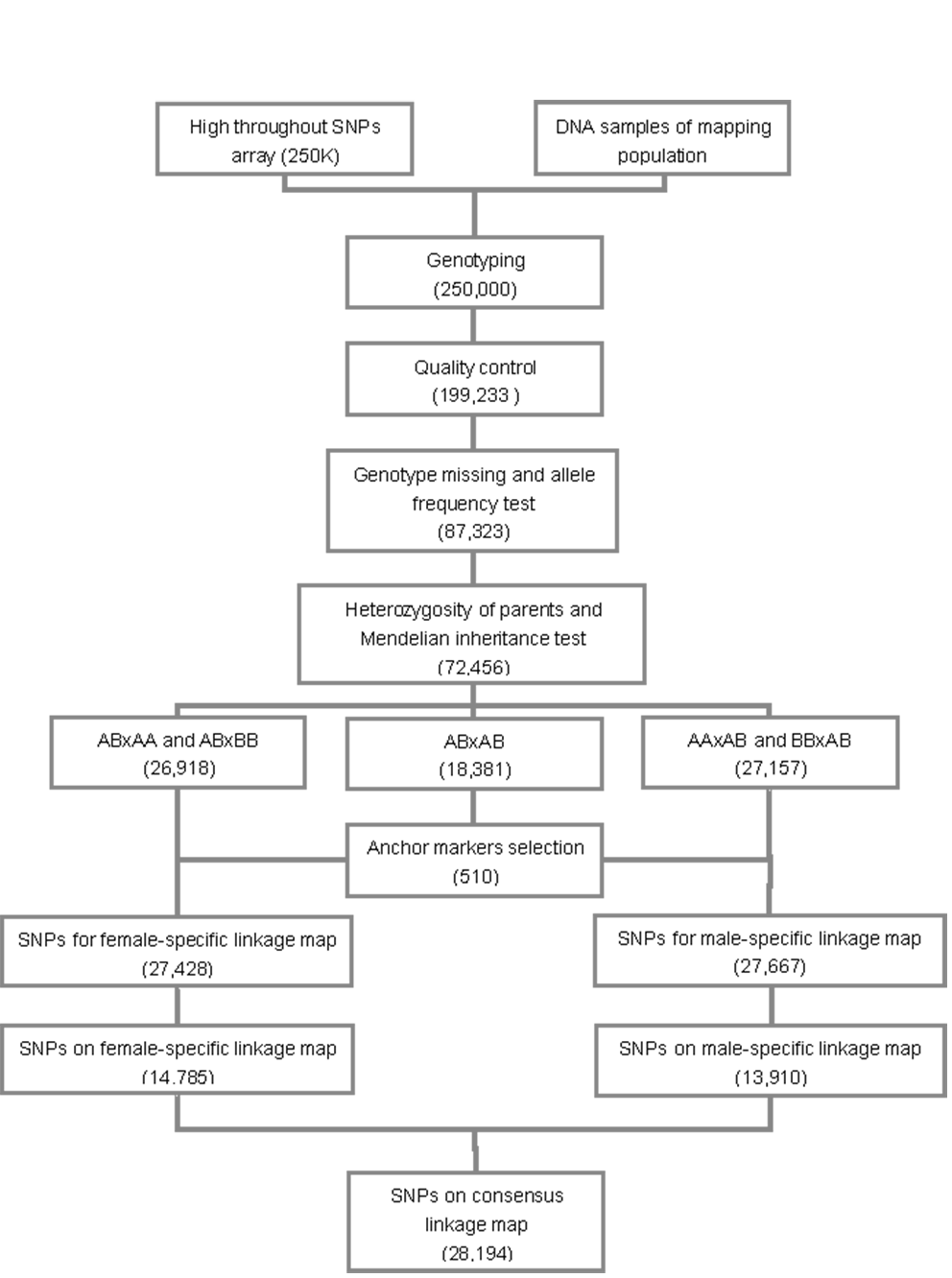


**Figure S2:** The female-specific linkage genetic map for Yellow River carp.


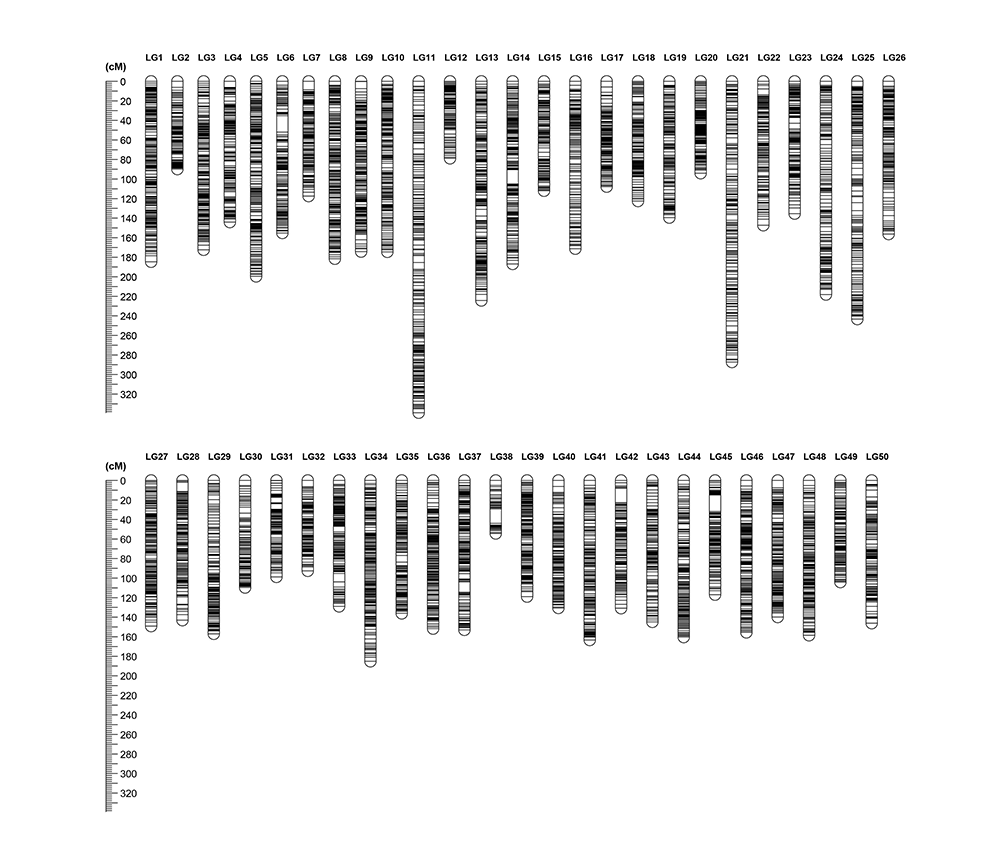


**Figure S3:** The male-specific linkage genetic map for Yellow River carp.


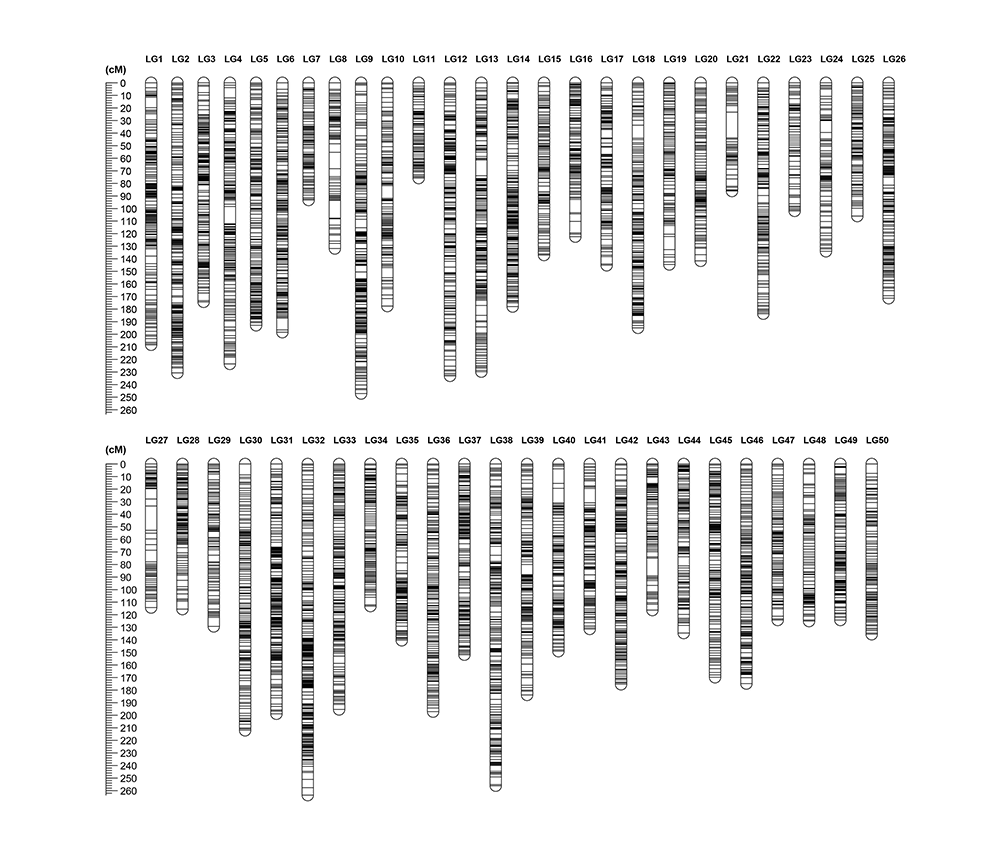


**Table S1.** Summary of the sex-specific linkage maps of Yellow River carp.

| LG | Female | | | | |  | Male | | | | |  | F:M ratio | | |
| --- | --- | --- | --- | --- | --- | --- | --- | --- | --- | --- | --- | --- | --- | --- | --- |
|  | No. of SNPs | Distinct positions | Distance (cM) | Locus interval  (cM) | Interval of shared markers (cM) |  | No. of SNPs | Distinct positions | Distance (cM) | Locus interval  (cM) | Interval of shared markers (cM) |  | Map length ratio | Locus  interval  ratio | Interval ratio  of shared markers |
| 1 | 407 | 209 | 184.62 | 0.89 | 21.86 |  | 338 | 192 | 208.37 | 1.09 | 16.44 |  | 0.89 | 0.81 | 1.33 |
| 2 | 164 | 96 | 89.75 | 0.94 | 8.10 |  | 412 | 213 | 230.67 | 1.09 | 10.13 |  | 0.39 | 0.87 | 0.80 |
| 3 | 384 | 190 | 172.43 | 0.91 | 15.26 |  | 302 | 170 | 174.17 | 1.03 | 14.47 |  | 0.99 | 0.89 | 1.05 |
| 4 | 312 | 143 | 144.04 | 1.01 | 18.31 |  | 420 | 213 | 223.60 | 1.05 | 21.86 |  | 0.64 | 0.96 | 0.84 |
| 5 | 381 | 198 | 199.58 | 1.01 | 18.87 |  | 349 | 180 | 192.87 | 1.08 | 16.07 |  | 1.03 | 0.94 | 1.17 |
| 6 | 287 | 133 | 155.17 | 1.18 | 13.82 |  | 472 | 191 | 198.43 | 1.04 | 16.28 |  | 0.78 | 1.13 | 0.85 |
| 7 | 245 | 131 | 117.54 | 0.90 | 11.91 |  | 214 | 104 | 93.22 | 0.91 | 7.80 |  | 1.26 | 1.00 | 1.53 |
| 8 | 416 | 208 | 181.62 | 0.88 | 16.00 |  | 141 | 73 | 131.75 | 1.83 | 10.49 |  | 1.38 | 0.48 | 1.53 |
| 9 | 393 | 187 | 174.32 | 0.94 | 16.99 |  | 404 | 229 | 247.13 | 1.08 | 17.09 |  | 0.71 | 0.86 | 0.99 |
| 10 | 378 | 203 | 174.52 | 0.86 | 14.69 |  | 247 | 127 | 177.58 | 1.41 | 8.69 |  | 0.98 | 0.61 | 1.69 |
| 11 | 448 | 227 | 339.13 | 1.50 | 9.14 |  | 141 | 82 | 75.77 | 0.94 | 7.90 |  | 4.48 | 1.60 | 1.16 |
| 12 | 202 | 94 | 78.77 | 0.85 | 7.20 |  | 376 | 217 | 233.08 | 1.08 | 9.55 |  | 0.34 | 0.78 | 0.75 |
| 13 | 466 | 224 | 224.31 | 1.01 | 25.06 |  | 383 | 210 | 229.71 | 1.10 | 17.45 |  | 0.98 | 0.92 | 1.44 |
| 14 | 367 | 180 | 186.90 | 1.04 | 15.51 |  | 420 | 205 | 178.01 | 0.87 | 17.63 |  | 1.05 | 1.20 | 0.88 |
| 15 | 248 | 126 | 112.04 | 0.90 | 11.00 |  | 215 | 121 | 137.01 | 1.14 | 5.29 |  | 0.82 | 0.79 | 2.08 |
| 16 | 257 | 133 | 171.32 | 1.30 | 4.17 |  | 233 | 108 | 122.42 | 1.14 | 7.08 |  | 1.40 | 1.13 | 0.59 |
| 17 | 268 | 124 | 107.80 | 0.88 | 10.19 |  | 241 | 125 | 145.20 | 1.17 | 11.68 |  | 0.74 | 0.75 | 0.87 |
| 18 | 287 | 131 | 122.51 | 0.94 | 9.28 |  | 326 | 183 | 194.92 | 1.07 | 13.04 |  | 0.63 | 0.88 | 0.71 |
| 19 | 275 | 138 | 139.51 | 1.02 | 14.17 |  | 209 | 111 | 144.46 | 1.31 | 10.89 |  | 0.97 | 0.78 | 1.30 |
| 20 | 253 | 121 | 93.96 | 0.78 | 8.19 |  | 228 | 123 | 141.65 | 1.16 | 7.03 |  | 0.66 | 0.67 | 1.17 |
| 21 | 397 | 187 | 287.24 | 1.54 | 2.92 |  | 76 | 48 | 86.08 | 1.83 | 3.97 |  | 3.34 | 0.84 | 0.74 |
| 22 | 276 | 134 | 147.35 | 1.11 | 12.33 |  | 311 | 167 | 183.64 | 1.11 | 13.45 |  | 0.80 | 1.00 | 0.92 |
| 23 | 264 | 142 | 135.35 | 0.96 | 13.35 |  | 124 | 64 | 101.91 | 1.62 | 7.02 |  | 1.33 | 0.59 | 1.90 |
| 24 | 320 | 157 | 218.15 | 1.40 | 5.22 |  | 153 | 79 | 134.03 | 1.72 | 3.99 |  | 1.63 | 0.81 | 1.31 |
| 25 | 371 | 190 | 243.28 | 1.29 | 4.51 |  | 223 | 103 | 105.96 | 1.04 | 4.91 |  | 2.30 | 1.24 | 0.92 |
| 26 | 311 | 154 | 156.27 | 1.02 | 15.83 |  | 322 | 189 | 171.26 | 0.91 | 16.33 |  | 0.91 | 1.12 | 0.97 |
| 27 | 376 | 175 | 149.10 | 0.86 | 17.14 |  | 127 | 63 | 114.25 | 1.84 | 12.23 |  | 1.31 | 0.47 | 1.40 |
| 28 | 264 | 142 | 143.02 | 1.01 | 12.62 |  | 232 | 122 | 115.53 | 0.95 | 8.87 |  | 1.24 | 1.06 | 1.42 |
| 29 | 248 | 140 | 157.06 | 1.13 | 12.43 |  | 192 | 94 | 129.33 | 1.39 | 7.93 |  | 1.21 | 0.81 | 1.57 |
| 30 | 212 | 91 | 109.57 | 1.22 | 7.78 |  | 334 | 171 | 212.00 | 1.25 | 13.21 |  | 0.52 | 0.98 | 0.59 |
| 31 | 224 | 96 | 98.73 | 1.04 | 11.35 |  | 407 | 235 | 198.77 | 0.85 | 18.40 |  | 0.50 | 1.22 | 0.62 |
| 32 | 216 | 102 | 92.58 | 0.92 | 6.97 |  | 454 | 263 | 263.63 | 1.01 | 12.00 |  | 0.35 | 0.91 | 0.58 |
| 33 | 256 | 131 | 128.89 | 0.99 | 11.60 |  | 373 | 206 | 195.37 | 0.95 | 16.40 |  | 0.66 | 1.04 | 0.71 |
| 34 | 353 | 209 | 185.14 | 0.89 | 16.69 |  | 192 | 111 | 113.08 | 1.03 | 10.12 |  | 1.64 | 0.87 | 1.65 |
| 35 | 267 | 148 | 136.10 | 0.93 | 14.12 |  | 254 | 129 | 140.38 | 1.10 | 12.56 |  | 0.97 | 0.84 | 1.12 |
| 36 | 321 | 171 | 151.71 | 0.89 | 12.69 |  | 341 | 175 | 197.12 | 1.13 | 16.34 |  | 0.77 | 0.79 | 0.78 |
| 37 | 350 | 154 | 152.89 | 1.00 | 18.12 |  | 336 | 169 | 151.81 | 0.90 | 17.54 |  | 1.01 | 1.11 | 1.03 |
| 38 | 76 | 36 | 54.27 | 1.55 | 5.35 |  | 437 | 223 | 256.07 | 1.15 | 9.15 |  | 0.21 | 1.34 | 0.58 |
| 39 | 276 | 155 | 118.95 | 0.77 | 12.40 |  | 297 | 172 | 183.84 | 1.08 | 12.18 |  | 0.65 | 0.72 | 1.02 |
| 40 | 282 | 131 | 130.52 | 1.00 | 10.28 |  | 259 | 131 | 149.03 | 1.15 | 10.45 |  | 0.88 | 0.88 | 0.98 |
| 41 | 367 | 157 | 163.35 | 1.05 | 13.10 |  | 253 | 126 | 131.28 | 1.05 | 10.51 |  | 1.24 | 1.00 | 1.25 |
| 42 | 235 | 116 | 130.80 | 1.14 | 10.82 |  | 320 | 178 | 175.38 | 0.99 | 15.23 |  | 0.75 | 1.15 | 0.71 |
| 43 | 237 | 116 | 144.81 | 1.26 | 8.80 |  | 177 | 90 | 116.33 | 1.31 | 7.75 |  | 1.24 | 0.96 | 1.14 |
| 44 | 309 | 153 | 160.36 | 1.05 | 14.05 |  | 234 | 113 | 134.62 | 1.20 | 13.21 |  | 1.19 | 0.88 | 1.06 |
| 45 | 197 | 114 | 117.01 | 1.04 | 10.39 |  | 293 | 160 | 169.77 | 1.07 | 14.46 |  | 0.69 | 0.97 | 0.72 |
| 46 | 301 | 162 | 155.52 | 0.97 | 12.65 |  | 316 | 163 | 174.78 | 1.08 | 11.11 |  | 0.89 | 0.90 | 1.14 |
| 47 | 252 | 149 | 139.88 | 0.95 | 12.28 |  | 186 | 103 | 124.16 | 1.22 | 9.49 |  | 1.13 | 0.78 | 1.29 |
| 48 | 315 | 165 | 158.49 | 0.97 | 12.52 |  | 172 | 89 | 125.15 | 1.42 | 8.44 |  | 1.27 | 0.68 | 1.48 |
| 49 | 231 | 110 | 104.11 | 0.96 | 8.52 |  | 246 | 124 | 124.22 | 1.01 | 9.74 |  | 0.84 | 0.95 | 0.88 |
| 50 | 243 | 121 | 146.24 | 1.22 | 11.11 |  | 198 | 110 | 135.53 | 1.24 | 8.23 |  | 1.08 | 0.98 | 1.35 |
| Total | 14785 | 7404 | 7586.51 | 1.02 | 12.15 |  | 13910 | 7347 | 8094.31 | 1.10 | 11.64 |  | 0.94 | 0.93 | 1.04 |

**Table S2.** Integration of consensus linkage map and reference genome of Songpu mirror carp.

| Item | Number | Percentage |
| --- | --- | --- |
| Number of SNPs on linkage map | 28194 | 100 |
| Number of SNPs mapped on genome scaffolds | 28194 | 100 |
| Number of SNPs used for anchoring genome scaffolds | 10119 | 35.9 |
| Number of genome scaffolds | 10783 | 100 |
| Number of genome scaffolds with SNP markers | 3212 | 29.8 |
| Number of genome scaffolds mapped onto different LGs | 1757 | 16.3 |
| Number of genome scaffolds anchored to linkage map | 2818 | 26.1 |
| Number of anchored scaffolds with at least two SNPs | 2325 | 21.6 |
| Total size of genome scaffolds (bp) | 1,713,798,505 | 100 |
| Total size of anchored genome scaffolds (bp) | 1,357,431,238 | 79.21 |

**Table S3.** Details of physical map of Songpu mirror carp anchored to linkage map.

| Linkage group | No. of SNPs | Total length of scaffolds (bp) |
| --- | --- | --- |
| LG1 | 267 | 37,205,647 |
| LG2 | 174 | 25,754,668 |
| LG3 | 212 | 29,393,492 |
| LG4 | 276 | 39,058,062 |
| LG5 | 277 | 33,196,892 |
| LG6 | 281 | 30,647,890 |
| LG7 | 193 | 26,560,361 |
| LG8 | 203 | 22,945,406 |
| LG9 | 288 | 43,185,573 |
| LG10 | 233 | 27,935,301 |
| LG11 | 198 | 27,545,178 |
| LG12 | 207 | 29,483,628 |
| LG13 | 307 | 36,023,240 |
| LG14 | 309 | 47,046,578 |
| LG15 | 161 | 22,266,198 |
| LG16 | 169 | 20,045,583 |
| LG17 | 165 | 25,046,199 |
| LG18 | 198 | 30,740,767 |
| LG19 | 184 | 28,205,903 |
| LG20 | 181 | 25,746,190 |
| LG21 | 145 | 18,532,797 |
| LG22 | 204 | 25,040,624 |
| LG23 | 114 | 17,108,631 |
| LG24 | 156 | 21,134,365 |
| LG25 | 223 | 28,215,066 |
| LG26 | 190 | 23,654,381 |
| LG27 | 174 | 28,154,536 |
| LG28 | 216 | 29,569,219 |
| LG29 | 116 | 13,419,470 |
| LG30 | 242 | 41,205,721 |
| LG31 | 203 | 25,736,468 |
| LG32 | 307 | 45,959,954 |
| LG33 | 244 | 31,535,479 |
| LG34 | 199 | 24,293,243 |
| LG35 | 155 | 16,573,744 |
| LG36 | 242 | 26,245,335 |
| LG37 | 258 | 34,445,723 |
| LG38 | 140 | 18,173,471 |
| LG39 | 220 | 25,908,263 |
| LG40 | 139 | 19,537,398 |
| LG41 | 260 | 36,576,071 |
| LG42 | 207 | 24,757,550 |
| LG43 | 136 | 16,569,016 |
| LG44 | 201 | 23,738,802 |
| LG45 | 149 | 31,129,771 |
| LG46 | 221 | 35,616,415 |
| LG47 | 118 | 12,952,252 |
| LG48 | 142 | 15,954,239 |
| LG49 | 180 | 20,426,922 |
| LG50 | 135 | 17,233,556 |
| Total | 10,119 | 1,357,431,238 |

**Table S4.** Correlation analysis of three growth-related traits in Yellow River carp.

| Traits | Items | BW | BL | CW |
| --- | --- | --- | --- | --- |
| BW | Pearson correlation | 1 | 0.845 | 0.930 |
|  | Sig.（Two-tailed） |  | 1.000E-013 | 1.000E-013 |
|  | No. of individuals | 103 | 103 | 97 |
| BL | Pearson correlation | 0.845 | 1 | 0.679 |
|  | Sig.(Two-tailed) | 1.000E-013 |  | 1.212E-013 |
|  | No. of individuals | 103 | 103 | 97 |
| CW | Pearson correlation | 0.930 | 0.679 | 1 |
|  | Sig.（Two-tailed） | 1.000E-013 | 1.212E-013 |  |
|  | No. of individuals | 97 | 97 | 98 |

**Table S5.** Integration of the consensus linkage map and reference genome of Hebao red carp.

| Item | Number | Percentage |
| --- | --- | --- |
| Number of SNPs on linkage map | 28,194 | 100 |
| Number of SNPs mapped on genome scaffolds | 26,308 | 93.3 |
| Number of SNPs used for anchoring genome scaffolds | 25,349 | 89.9 |
| Number of genome scaffolds | 625,057 | 100 |
| Number of genome scaffolds mapped onto different LGs | 124 | 0.02 |
| Number of genome scaffolds anchored to linkage map | 2,963 | 0.5 |
| Number of anchored scaffolds with at least two SNPs | 1690 | 0.27 |
| Total size of genome scaffolds (bp) | 1,538,508,504 | 100 |
| Total size of anchored genome scaffolds (bp) | 1,236,461,539 | 80.4 |

**Table S6.** Details of physical map of Hebao red carp anchored to linkage map.

| Linkage group | No. of SNPs | Total length of scaffolds (bp) |
| --- | --- | --- |
| LG1 | 676 | 30,539,024 |
| LG2 | 509 | 29,879,942 |
| LG3 | 595 | 27,382,184 |
| LG4 | 664 | 26,136,356 |
| LG5 | 638 | 26,652,056 |
| LG6 | 672 | 30,935,915 |
| LG7 | 392 | 18,466,758 |
| LG8 | 497 | 24,153,890 |
| LG9 | 716 | 35,073,684 |
| LG10 | 557 | 33,212,892 |
| LG11 | 508 | 27,328,810 |
| LG12 | 521 | 26,465,823 |
| LG13 | 763 | 35,624,239 |
| LG14 | 688 | 35,132,079 |
| LG15 | 416 | 24,844,494 |
| LG16 | 445 | 23,976,800 |
| LG17 | 447 | 26,964,254 |
| LG18 | 551 | 27,332,841 |
| LG19 | 425 | 20,139,439 |
| LG20 | 410 | 20,399,690 |
| LG21 | 416 | 20,001,548 |
| LG22 | 529 | 22,554,982 |
| LG23 | 336 | 22,661,456 |
| LG24 | 424 | 21,957,121 |
| LG25 | 530 | 27,026,268 |
| LG26 | 562 | 25,191,882 |
| LG27 | 450 | 23,622,845 |
| LG28 | 444 | 22,946,105 |
| LG29 | 387 | 23,329,123 |
| LG30 | 473 | 22,136,666 |
| LG31 | 560 | 26,854,179 |
| LG32 | 593 | 24,776,003 |
| LG33 | 553 | 25,697,785 |
| LG34 | 472 | 24,063,976 |
| LG35 | 445 | 23,131,649 |
| LG36 | 586 | 26,066,699 |
| LG37 | 609 | 25,973,395 |
| LG38 | 447 | 24,057,966 |
| LG39 | 492 | 24,177,598 |
| LG40 | 474 | 24,972,799 |
| LG41 | 547 | 22,481,537 |
| LG42 | 491 | 20,861,614 |
| LG43 | 366 | 17,552,105 |
| LG44 | 483 | 20,645,866 |
| LG45 | 429 | 21,490,787 |
| LG46 | 536 | 22,368,071 |
| LG47 | 383 | 21,414,174 |
| LG48 | 426 | 22,080,483 |
| LG49 | 422 | 16,977,888 |
| LG50 | 394 | 18,747,799 |
| Total | 25,349 | 1,236,461,539 |
